# Distinct Gene Expression Patterns of Two Heat Shock Protein 70 Members During Development, Diapause, and Temperature Stress in the Freshwater Crustacean *Daphnia magna*

**DOI:** 10.3389/fcell.2021.692517

**Published:** 2021-07-01

**Authors:** Luxi Chen, Rocío Gómez, Linda C. Weiss

**Affiliations:** ^1^Department of Animal Ecology, Evolution and Biodiversity, Ruhr-University Bochum, Bochum, Germany; ^2^Departamento de Biología, Facultad de Ciencias, Universidad Autónoma de Madrid, Madrid, Spain

**Keywords:** Hsp70, *Daphnia*, diapause, embryonic development, temperature stress

## Abstract

Dormancy is a lifecycle delay that allows organisms to escape suboptimal environmental conditions. As a genetically programmed type of dormancy, diapause is usually accompanied by metabolic depression and enhanced tolerance toward adverse environmental factors. However, the drivers and regulators that steer an organism’s development into a state of suspended animation to survive environmental stress have not been fully uncovered. Heat shock proteins 70 (HSP70s), which are often produced in response to various types of stress, have been suggested to play a role in diapause. Considering the diversity of the Hsp70 family, different family members may have different functions during diapause. In the present study, we demonstrate the expression of two *hsp70* genes (A and B together with protein localization of B) throughout continuous and diapause interrupted development of *Daphnia magna*. Before and after diapause, the expression of *Dmhsp70-A* is low. Only shortly before diapause and during diapause, *Dmhsp70-A* is significantly upregulated and may therefore be involved in diapause preparation and maintenance. In contrast, *Dmhsp70-B* is expressed only in developing embryos but not in diapausing embryos. During continuous development, the protein of this Hsp70 family member is localized in the cytosol. When we expose both embryo types to heat stress, expression of both *hsp70* genes increases only in developing embryos, and the protein of family member B is translocated to the nucleus. In this stress formation, this protein provides effective protection of nucleoplasmic DNA. As we also see this localization in diapausing embryos, it seems that *Daphnia* embryo types share a common subcellular strategy when facing dormancy or heat shock, i.e., they protect their DNA by HSP70B nuclear translocation. Our study underlines the distinctive roles that different Hsp70 family members play throughout continuous and diapause interrupted development.

## Introduction

Members of different species groups ranging from invertebrates to vertebrates can overcome harsh environments using a dedicated strategy called dormancy, in which development and metabolism are suppressed (Denlinger, 2002; Hand et al., 2016). Diapause is a genetically programmed form of dormancy and requires adaptive physiological changes by which severe environmental conditions are evaded. During diapause, a state of metabolic arrest is entered upon which the organism becomes highly resistant to environmental stressors (Rinehart et al., 2006, 2007; MacRae, 2010; Kankare et al., 2016; Tan and MacRae, 2018; Wagner et al., 2018). In arthropods, for example, a 100% survival rate was found in diapausing pupae of the oak silkmoth *Antheraea pernyi* after 1 h exposure to −15°C, whereas only ∼4% of non-diapausing survived the same exposure (Liu et al., 2016).

Stress tolerance during diapause is discussed to be achieved *via* a variety of diapause-unique physiological and biochemical mechanisms (Denlinger, 2002; Rozsypal et al., 2013; Hand et al., 2016; Diniz et al., 2017). For example, heat shock proteins (HSPs) have been shown to function in stress responses in a range of species (see Table 1). HSPs function as molecular chaperones, protect cellular proteins from stress-induced denaturation, and thereby enhance cellular resistance against different environmental stressors (Feder and Hofmann, 1999; Jolly and Morimoto, 2000). The different types of HSPs are classified based on their molecular weight into different families. The most common examples are members of the Hsp23, Hsp60, Hsp70, and Hsp90 families (Li and Srivastava, 2004; Datta et al., 2017). Upon these, Hsp70 is probably the best studied (Denlinger et al., 2001). Proteins of this gene family have a molecular weight of approximately 70 kDa and represent one of the most conserved *hsp* gene families among species. The encoded HSP70 proteins can engage protein folding and translocation but are also involved in other vital functions such as signal transduction and cell cycle (Luft and Dix, 1999; Schumpert et al., 2014; Radons, 2016; Rosenzweig et al., 2019). Although some HSP70 proteins are constantly expressed, others are stress-inducible (Radons, 2016).

**TABLE 1:** An overview of expression of Hsp70 family during diapause in different species.

Stage of diapause	Species	Expression during diapause	References
Embryonic diapause	*Lymantria dispar* (Gypsy Moth)	Upregulated	Rinehart et al., 2007
*Austrofundulus limnaeus* (annual killifish)	Podrabsky and Somero, 2007	
*Bombyx mori* (silkworm)	Moribe et al., 2010; Sasibhushan et al., 2013	
	
	*Bombyx mori* (silkworm)	Constant	Saravanakumar et al., 2008
	*Allonemobius socius* (ground cricket)		Reynolds and Hand, 2009

Pupal diapause	*Sarcophaga crassipalpi*s (flesh fly)	Upregulated	Rinehart et al., 2000, 2007, 2010; Hayward et al., 2005; Li et al., 2007; Ragland et al., 2010
	*Rhagoletis pomonella* (apple maggot fly)		Rinehart et al., 2007
	*Rhagoletis suavis* (walnut husk maggot)		Rinehart et al., 2007
	*Megachile rotundata* (solitary bee)		Yocum et al., 2005
	*Manduca sexta* (tabacco hornworm)		Rinehart et al., 2007
	*Delia antiqua* (onion maggot)		Chen et al., 2006; Hao et al., 2012
	*Helicoverpa armigera* (cotton bollworm)		Bao and Xu, 2011
	
	*Helicoverpa zea* (corn earworm)	Constant	Zhang and Denlinger, 2010

Larval diapause	*Calliphora vicina* (blow fly)	Upregulated	Fremdt et al., 2013
	*Ostrinia nubilalis* (european corn borer)		Rinehart et al., 2007
	
	*Lucilia sericata* (blow fly)	Constant	Tachibana et al., 2005
	*Sesamia nonagrioides* (corn stalk borer)		Gkouvitsas et al., 2009

Juvenile diapause	*Calanus finmarchicus*	Downregulated	Aruda et al., 2011
	
	*Calanus finmarchicus*	Constant	Aruda et al., 2011

Reproductive diapause	*Drosophila triauraria* (fruit fly)	Constant	Goto et al., 1998
	*Leptinotarsa decemlineata* (Colorado potato beetle)		Yocum, 2001
	*Culex pipiens* (northern hose mosquito)		Rinehart et al., 2006
	
	*Leptinotarsa decemlineata* (Colorado potato beetle)	Upregulated	Yocum, 2001

Based on these manifold functions, Denlinger et al. (2001) hypothesized that certain HSP70 proteins participate in developmental arrest and cell cycle regulation during diapause. Rinehart et al. (2007) discussed that one member of the Hsp70 family enhances stress tolerance during diapause but does not control diapause itself. These assumptions are further complicated because different species show differential expression of various Hsp70 family members during diapause (Table 1). Among them, upregulation of *hsp70* genes was reported in diapausing flies *Sarcophaga crassipalpis* (Rinehart et al., 2000, 2007) and *Delia antiqua* (Hao et al., 2012); however, downregulation was reported in the potato beetle *Leptinotarsa decemlineata* (Yocum, 2001) and the crustacean *Calanus finmarchicus* (Aruda et al., 2011). One reason for these different observations could be that previous studies investigated different Hsp70 family members and that the different *hsp70* genes could play different roles in diapause.

We here aimed to obtain a first insight into the involvement of two *hsp70* genes during diapause in the model freshwater crustacean *Daphnia magna*. *Daphnia* can switch between asexual and sexual reproduction in response to environmental conditions. When the conditions are favorable, *Daphnia* females produce high numbers of parthenogenetic embryos. These asexually produced embryos complete their development in the mother’s brood pouch and are released as fully developed juveniles (i.e., direct embryogenesis; Decaestecker et al., 2009). When the conditions decline, for example, with the shortening of photoperiod, changes in temperature, an increase in the population’s density, reduction in food quantity and quality, or even the presence of predators, *Daphnia* females switch to sexual reproduction (Stross and Hill, 1965; Hobaek and Larsson, 1990; Kleiven et al., 1992; Alekseev and Lampert, 2001; Ślusarczyk et al., 2005; Koch et al., 2009). Then, most *Daphnia* species produce genetically identical males and haploid oocytes (Bull, 1985; Hiruta and Tochinai, 2014). Upon fertilization of the oocytes through the male’s spermatozoa, these sexually produced embryos will develop under the protection of a robust shell called the ephippium (Pancella and Stross, 1963). Development ceases even before the ephippium is cast off during the next molting cycle of the *Daphnia* mother (Chen et al., 2018). Encapsulated in their ephippia, the diapausing embryos sometimes first float on the water surface or directly sink to the sediment where they can arrest in diapause for years up to decades (Pietrzak and Ślusarczyk, 2006; Ślusarczyk and Pietrzak, 2008; Frisch et al., 2014; Cambronero and Orsini, 2017). In this state of suspended animation, the embryos become highly resistant to harsh conditions, for instance, extreme temperatures (−84 and + 110°C) (Radzikowski, 2013). Once environmental conditions become habitable again, embryos are resurrected by exogenous factors (e.g., light) and then develop into juveniles that hatch from the ephippia to repopulate the habitat (Decaestecker et al., 2009). In this case, embryogenesis is interrupted by a diapause phase.

**FIGURE 1:**
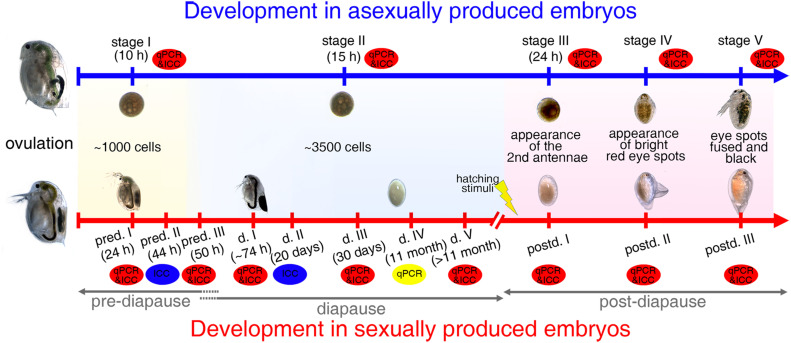
Embryogenesis in *D. magna* asexually and sexually produced embryos. Representative and comparative sampling stages collected for qPCR and immunolabeling experiments: “stage I” asexually produced embryos and “pred. I” sexually produced embryos contain ∼1,000 cells; “pred. II” embryos are collected 44-h post-ovulation; “stage II,” “pred. III,” and “d. I/II/III/IV/V” embryos contain ∼3,500 cells; “stage III” and “postd. I” embryos are collected when second antennae are formed; “stage IV” and “postd. II” embryos have two bright red-eye spots; “stage V” and “postd. III” embryos are determined based on formation of one black compound eye. pred.: pre-diapause; d.: diapause; postd.: post-diapause.

In general, different members of the Hsp70 family contribute significantly to stress tolerance in both non-diapausing and diapausing organisms. We, therefore, hypothesized the involvement of Hsp70 in enhancing stress resistance in *D. magna* diapausing embryos. For a first insight, we focused on two Hsp70 members: *Dmhsp70-A* (Gene ID: Dapma7bEVm002643t1) and *Dmhsp70-B* (Gene ID: Dapma7bEVm003127t13). Gene expression of these two *hsp70*s was monitored using quantitative polymerase chain reaction (qPCR), whereas the subcellular localization of HSP70B was determined, capitalizing on an antibody detecting the gene products of *Dmhsp70-B*. Unfortunately, there is no antibody detecting the gene products of *Dmhsp70-A*. We examined whether the two *hsp70* genes are differentially expressed during five stages of *Daphnia* continuous and nine stages of diapause interrupted embryogenesis (Figure 1). We then surveyed whether the two *hsp70* genes can respond to temperature stress (heat and cold) during diapause. In line with this, we immunocytochemically determined the subcellular localization of HSP70B in both types of embryogenesis and under stress conditions. To our knowledge, this is the first study that shows the expressions of different Hsp70 members throughout the embryogenesis of continuously developing and diapause destined embryos of *D. magna*.

## Materials and Methods

### Animal Culture

Continuously developing embryos were collected from parthenogenetically reproducing *D. magna* clone FT442 (from Finland and kindly provided by Dieter Ebert). Animals were kept at a density of six individuals/liter with a long-day photoperiod (16:8-h light:dark cycle) in 1-L glass jars (WECK^®^, Germany). As diapause in *Daphnia* is always associated with sexual reproduction, we used embryos from crosses of the *D. magna* clone FT442 and *D. magna* clone Elias (from Lake Elias, Egypt, collected by L. Weiss and R. Tollrian). Both *D. magna* clones have been raised in our laboratory since 2013 in a clone-specific manner. To induce male production, clone Elias was kept under short-day photoperiod (8:16-h light:dark cycle) at a density of 20 individuals/liter. Thirty females of clone FT442, together with 20 males of clone Elias, were then raised in one 1-L glass jar (WECK, Germany) under short-day photoperiod. All animals were cultured in an artificial *Daphnia* medium (ADaM, Klüttgen et al., 1994) at 20 ± 0.1°C and fed with the algae *Acutodesmus obliquus ad libitum* > 1.5 g carbon/L. The remnants and exuvia were removed daily using glass pipettes, and the culture medium was exchanged weekly.

### Stage-Specific Sampling Procedure

For qPCR, we collected a total of 30 animals in three replicates per stage and treatment and prepared 15 individuals for immunocytochemistry per treatment and stage.

To determine differential expression patterns of two *hsp70* genes during the continuous and diapause interrupted embryonic development of *D. magna*, we selected dedicated developmental stages before diapause, during diapause, and after the resurrection. These were chosen based on cell number that the animals reach at dedicated points in time post-ovulation (when raised at 20 ± 0.1°C). Points in time were selected based on previously established cell number curves over time for both embryo types (Chen et al., 2018). Successful resurrection was determined with the help of explicit morphological features (i.e., the appearance of the antennal buds, red and black eyes; Schwarzenberger et al., 2020). We chose five representative stages during embryonic development in *D. magna* asexually produced embryos and nine comparable stages in sexually developing embryos (Figure 1).

#### Collection of Asexually Produced Embryos

In asexually produced embryos, we monitored ovulation as reported previously (Chen et al., 2018). Briefly, females from *D. magna* clone FT442 with filled ovaries were cultured individually in 50-ml snap cap vials containing 40-ml ADaM and *A. obliquus ad libitum* at 20 ± 0.1°C with a long photoperiod (16:8 h). The moment of ovulation was checked every 15 min. “Stage I” embryos with ∼1,000 cells were collected at 10-h post-ovulation. ‘Stage II’ embryos were collected 15-h post-ovulation and had ∼3,500 cells. “Stage III” embryos were collected when the second antennae and the abdominal appendages were formed. “Stage IV” embryos had two bright red-eye spots, which fused to one compound black eye in “stage V” embryos (Figure 1).

#### Collection of Sexually Produced Embryos

The sexually produced embryos were cultured identically to asexually produced embryos. One female *D. magna* clone FT442 with condensed ovaries indicating the presence of one to two haploid embryos was co-cultured with one extra male *D. magna* clone Elias under short photoperiod (8:16 h) conditions. Again, the moment of ovulation was checked every 15 min.

We divided embryonic development in sexually produced *D. manga* embryos into three phases: pre-diapause (pred.), diapause (d.), and post-diapause (postd.; Figure 1). The pre-diapause stages were collected according to dedicated time points post-ovulation: we collected embryos at 24-h (“pred. I,” embryos with ∼1,000 cells), 44-h (“pred. II”), and 50-h (“pred. III,” embryos with ∼ 3500 cells) post-ovulation when the ephippia were not yet cast off.

The beginning of diapause is defined as developmental arrest (Koštál, 2006). We used the mitotic activity to define diapause entrance, ∼50-h post-ovulation (Chen et al., 2018). When the ephippia were cast off (∼74-h post-ovulation), they were either collected as diapause stage I embryos (“d. I”) or transferred into Eppendorf tubes with a drop of sterile ADaM medium and stored at 4 ± 0.1°C shielded from light in diapause conditions. After either 20 days (“d. II”), 30 days (“d. III”), 11 months (“d. IV”), or > 11 months (“d. V”) at the conditions as mentioned earlier, the diapausing embryos were collected.

Post-diapause stages were defined as the developmental phases when diapausing embryos were exposed to hatching stimuli and selected morphological features are observable. To induce resurrection, diapausing embryos were dissected from the ephippia with fine-tipped metal tweezers after a diapause length of 30 days. The embryos were transferred into sterile 24-well plates (VWR, Germany) filled with 2-ml sterile ADaM and exposed to constant photoperiod (simulated by fluora and biolux lamps, Osram, Germany) at 23 ± 0.1°C (Chen et al., 2018). The hatching success was checked daily using a stereomicroscope (SZX12, Olympus). Sterile ADaM medium was refreshed every 2 days. “Postd. I,” “postd. II,” and “postd. III” embryos are morphologically comparable with the “stage III,” “stage IV,” and “stage V” asexually produced embryos (Figure 1).

When the embryos reached the respective stages, they were removed from the ephippium or maternal brood pouch using fine forceps. Embryos were either fixed in freshly prepared 4% PFA-TX [formaldehyde 37%; Merck, Germany; diluted in phosphate-buffered saline (PBS) 0.1 M, pH 7.4; with 0.05% Triton X; Serva, Germany] for immunolabeling or were transferred to 1.5-ml Eppendorf tubes (Sarstedt, Germany) and snap-frozen in liquid nitrogen and stored at −80 ± 0.1°C until RNA extraction. All samples were collected and processed within the same period between August and November 2020.

### Effect of Diapause-Maintenance Temperature on *hsp70* Gene Expression

In nature, *Daphnia* diapause is often associated with low environmental temperatures, so that these natural conditions are mimicked and the diapausing embryos are stored at four ± 0.1°C (De Meester and De Jager, 1993; Haghparast et al., 2012; Navis et al., 2015; Mushegian et al., 2018; Radzikowski et al., 2018). Here, we wanted to exclude the impact of the low diapause-maintenance temperature on the expression of Hsp70 targets. For this, we analyzed the expression patterns of both *hsp70* genes in diapausing embryos that were kept at 20 ± 0.1°C, which alone has no obvious effect (i.e., it does not initiate development) on diapause maintenance (Schwartz and Hebert, 1987; Chen et al., 2018). After the ephippia were cast off, they were handled likewise but stored at 20 ± 0.1°C (and not 4 ± 0.1°C) in darkness. After 30 days and > 11 months, diapausing embryos were collected for gene expression analysis. The gene expression level was compared with the embryos with the same diapause length stored at 4 ± 0.1°C.

### *D. magna* Embryo Exposure to Temperature Stress

To assess whether Hsp70 members are temperature-inducible, we exposed sexually produced diapausing embryos and asexually produced developing embryos to hot and cold conditions. *Hsp70* messenger RNA (mRNA) expression is not heat-inducible before midblastula in *Xenopus laevis* embryos (Lang et al., 2000) or before the 12th cell cycle in *Drosophila* embryos (Wang and Lindquist, 1998). For a significant stress response in active cells, “stage III” asexually produced embryos were used rather than the earlier embryonic stages (“stage I” and “stage II”). Temperature stress on embryos was performed by collecting pregnant *D. magna* FT442 females with “stage III” asexually produced embryos in their brood chamber raised at 20 ± 0.1°C. Based on our preliminary experiments, 3 ± 0.1°C and 33 ± 0.1°C were chosen, as these temperatures did not affect animal survival (see Supplementary Figure 1A). The mothers were transferred individually into 1.5-ml Eppendorf tubes filled with 1-ml ADaM. One hour cold shock was performed on ice, so that the final temperature of ADaM reached 3 ± 0.1°C (determined using a digital thermometer, Amarell GmBH, Germany). Heat shock was performed by again transferring pregnant mothers with “stage III” asexually produced embryos into 1.5-ml Eppendorf tubes using Thermocell MixingBlock (Bioer MB-102). In the maternal brood pouch, the asexually produced embryos were heat-exposed at 33 ± 0.1°C for 1 h. Temperatures were validated with a digital thermometer. Control group embryos were performed likewise but in a climate cabinet at 20 ± 0.1°C.

Embryos in diapause were stressed in a similar manner but with different temperatures. Mid diapause sexually produced embryos that had been stored for 30 days at 4 ± 0.1°C were then exposed to temperature stress. We observed hatching events of control and stressed embryos after exposing the embryos to the hatching conditions described earlier. To ensure a strong enough temperature stress, sexually produced embryos were exposed to −20 ± 0.1°C (freezer) and 40 ± 0.1°C (in a Thermocell MixingBlock; temperature was checked with a digital thermometer; see Supplementary Figure 1B). For stress exposure, diapausing embryos were left in the intact ephippia. All stress conditions were shielded from light. Control groups were handled accordingly, but embryos remained in darkness at 4 ± 0.1°C.

Samples were collected either directly after temperature stress and after a recovery period of 2 or 4 h (sexually produced embryos: 4 ± 0.1°C and dark; asexually produced embryos: 20 ± 0.1°C and light). Subsequently, the embryos were removed from the females or ephippia and prepared for downstream immunolabeling and gene expression analysis.

### Immunocytochemistry

In this study, we used a polyclonal HSP70 antibody raised in rabbits (10995-1-AP, Proteintech, Germany). Protein sequence of the used antibody shares homology with the gene product of *Dmhsp70-B* (84%) but only 46% homology with the gene product of *Dmhsp70-A.* Therefore, it is highly unlikely that the antibody used stains proteins produced by *Dmhsp70-A.* To better identify the cellular distribution of HSP70B protein, phalloidin (sc-363795, Santa Cruz, Germany) was used to stain the cytoskeletal component, i.e., actin microfilament networks, and determine the size and shape of cells as this changes during diapause progression (Chen et al., 2018). Immunolabeling was performed as published previously (Chen et al., 2018). Briefly, after 15-min fixation in 4% PFA-TX, the embryos were squashed with coverslips on poly-lysine-coated object slides (VWR, Germany) and immersed in PBS (0.1 M, pH 7.4). The coverslips were flipped off with a razor blade. The slides were rinsed with PBS and incubated with the HSP70-antibody diluted 1/60 in PBS for 2 h at room temperature. After three 5-min washes in PBS, goat anti-rabbit IgG (Alexa 488, Dianova Germany, dilution: 1/120 in PBS) together with phalloidin (dilution: 1/90 in PBS) were applied to detect the primary antibody and F-actin. Slides were incubated in the dark for 1 h at room temperature and subsequently rinsed three times in PBS for 5 min, mounted, and cover-slipped in DAPI-Vectashield (H-120, Vecta Laboratories, Burlington, VT, United States). The coverslips were fixed and sealed with rapidly solidifying nail varnish; preparations were kept in the dark at 4 ± 0.1°C until imaging.

### Image Processing

Immunolabeling images were documented using a Zeiss Axiophot fluorescent microscope equipped with an Olympus XC10 monochrome digital camera controlled by the software CellSense (Olympus, Germany). The contrast and brightness of the immunofluorescence image were adjusted and assembled using Adobe Photoshop CS6.

### Gene Expression Patterns

To measure *hsp70* changes on the gene level during development or temperature stresses, we determined differential mRNA expression of *Dmhsp70-A* and *Dmhsp70-B* using qPCR.

#### Sample Collection and RNA Extraction

We collected three biological samples of each developmental stage with 30 individuals per sample. Embryos were manually homogenized using a sterile pestle, RNA was isolated with the ReliaPrep RNA Miniprep system (Promega, Germany) for tissues according to the manufacturer’s protocol. The RNA quality was checked using an Eppendorf BioPhotometer (Eppendorf, Germany). The RNA integrity index was determined using an Agilent Bioanalyzer microchip reader (Agilent, Germany) and an RNA 6000 nano kit. Samples with a RNA integrity number > 8.0 were used. RNA concentration was assessed using Qubit RNA broad range assay kit (Thermo Fisher Scientific, Germany).

#### Primer Design and Validation

Primer pairs for the gene *Dmhsp70-A*, *tbp* (Tata-box binding protein), and *L32* (a ribosomal protein) were obtained from Heckmann et al. (2006); Kato et al. (2007), and Imhof et al. (2017). *Dmhsp70-B* primer pairs were designed based on the protein sequence of Dapma7bEVm003127t13 using Primer3^1^ (Supplementary Table 1) (Table 2). We chose this specific transcript, as its sequence best matches the protein sequence targeted by the applied HSP70 antibody.

**TABLE 2:** qPCR primer pairs used in RT-qPCR.

Gene name	Abbreviation	Gene ID/wfleaBase	Primer forward (5′–3′)	Primer reverse (5′–3′)	T_melt_	Amplicon size (bp)	References
*Heat shock protein 70*	*Dmhsp70-A*	Dapma7bEV m002643t1	TGTCCAGACTTA CC ATAAGCA	CAACGTCAAGCA ACAAAGGA	60°C	103	Imhof et al., 2017
*Heat shock protein 70*	*Dmhsp70-B*	Dapma7bEV m003127t13	GCTCGTTTCGAA GAGTTGAATGCC	TCCTAGTTGAAC CACCAACAAGAACA	60°C	130	This study
*Ribosomal protein*	*L32*	Dapma7bEV m010195	GACCAAAGGGTA TTGACAACAGA	CCAACTTTTGGC ATAAGGTACTG	60°C	100	Kato et al., 2007; Nong et al., 2020
*Tata-box binding protein*	*tbp*	WFes0002485	GCAGGGAAGTTT AGTTTCTGGA	TGGTATGCACAG GAGCAAAG	60°C	88	Heckmann et al., 2006

#### Reverse Transcription Quantitative Polymerase Chain Reaction

qPCR was performed with the Luna Universal One-Step Reverse Transcription qPCR kit (New England Biolabs, Germany) in a Light Cycler96 (Roche, Germany) as described by the manufacturer’s protocol. Ninety-six-well plates (Sarstedt, Germany) were set up in technical duplicates and three biological replicates, including non-template controls (RNA replaced with water) as well as reverse transcription controls (reverse transcriptase was substituted with water). qPCR reaction was performed in a total reaction volume of 10 μl, including 1 μl of 10 ng/μl RNA, 0.8 μl of 10-μM gene-specific primer pairs (Table 2), 5-μl reaction mix (2×), 0.5-μl RT enzyme mix, and 2.7-μl nuclease-free water. The plates were incubated at 55°C for 600 s and 95°C for 60 s, followed by 45 cycles at 95°C for 10 s and at 60°C for 30 s. A melting curve analysis was performed after the amplification to confirm the specificity of the PCR products. We used a total of nine plates and ensured comparability between plates by including two standard RNA samples that we amplified and were run with the reference gene *tbp* and *L32* on every plate as a standard.

#### Statistical Analyses for Reverse Transcription Quantitative Polymerase Chain Reaction

We used RefFinder to ensure the stability of the selected reference genes between all treatments, stages, and sexually and asexually produced embryo types (Xie et al., 2012). *Tbp* and *L32* were stably expressed over all embryonic stages irrespective of temperature shocks and thus were used as reference genes (Supplementary Table 2). The primer efficiency was determined for each qPCR reaction by calculating the slope of the linear phase using the software “LinReg” (Ruijter et al., 2009). Target gene expression was normalized to both of the selected reference genes according to Pfaffl in REST (Pfaffl, 2001). Gene expression of both Hsp70 members was normalized to a standard stage and log2-transformed. Results were presented using R (R Core Team, 2020) within RStudio (RStudio Team, 2020), generated with the packages “ggplot2” (Wickham, 2016) and “ggalt” (Rudis et al., 2017).

## Results

This study aimed to identify the expression changes of two Hsp70 family members on the gene and protein level before, during, and after *D. magna* diapause in comparison with continuously developing asexually produced embryos. Subsequently, we investigated the stress inducibility of these two Hsp70s to determine their putative role in temperature resistance.

### Gene Expression of *Dmhsp70-A* and *Dmhsp70-B* During Embryonic Development in Asexually Produced Embryos

**FIGURE 2:**
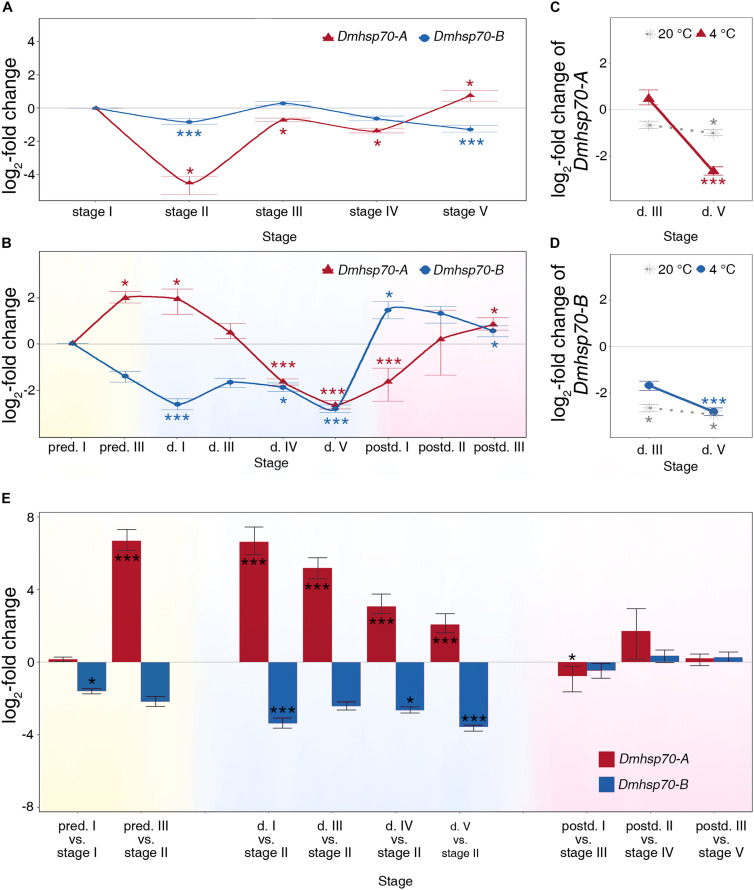
Relative fold change (in log_2_ scale) in transcript level of *Dmhsp70-A* and *Dmhsp70-B. Dmhsp70-A* and *Dmhsp70-B* during embryogenesis of asexually produced embryos **(A)** and sexually produced embryos **(B)**. Displayed is expression at each developmental stage relative to first stage (“stage I” in asexually produced embryos and “pred. I” in sexually produced embryos). *Dmhsp70-A* **(C)** and *Dmhsp70-B* **(D)** in 30-day-old diapausing embryos, which were stored at 20 ± 0.1°C. Displayed is expression at each developmental stage relative to “pred. I.” Comparison of gene expression of *Dmhsp70-A* and *Dmhsp70-B* (in log_2_ scale) between asexually and sexually produced embryos **(E)**. Error bars represent standard error (n = 3). *P*-values are indicated by asterisks: *P ≤ 0.05, ***P ≤ 0.005.

Fold change in gene expression and *P*-values are listed in Supplementary Table 3. We find a similar expression pattern of *Dmhsp70-A* and *Dmhsp70-B* during asexual embryogenesis. In comparison with “stage I” asexually produced embryos, the relative expression of *Dmhsp70-A* is significantly downregulated at “stage II,” “stage III,” and “stage IV.” “Stage V” has a significantly higher *Dmhsp70-A* expression level compared with “stage I” (Figure 2A). *Dmhsp70-B* expression is significantly downregulated at “stage II” in comparison with “stage I.” Gene expression is stable at “stage III” and “stage IV” compared with “stage I” and significantly downregulated at “stage V” compared with “stage I” (Figure 2A). The expression patterns of *Dmhsp70-A* and *Dmhsp70-B* are quite similar, whereas *Dmhsp70-B* shows a more stable expression profile.

### Gene Expression of *Dmhsp70-A* and *Dmhsp70-B* During Embryonic Development in Sexually Produced Embryos

The expression pattern of *Dmhsp70-A* and *Dmhsp70-B* is completely different throughout diapause development in *D. magna* sexually produced embryos. During the pre-diapause stage in sexually produced embryos, gene expression of *Dmhsp70-A* is increased when comparing “pred. III” with “pred. I.” When the embryos enter diapause, *Dmhsp70-A* expression remains high at “d. I” in comparison with “pred. I.” In 30-day-old diapausing embryos (“d. III”), gene expression of *Dmhsp70-A* equals with the gene expression at “pred. I.” This level is significantly decreased at “d. IV” and “d. V” in comparison with “pred. I.” When the embryonic development resumes, *Dmhsp70-A* gene expression at “postd. I” is significantly lower than “pred. I.” After the continuous embryonic development, its expression levels are higher than “pred. I” (Figure 2B). During pre-diapause, *Dmhsp70-B* gene expression at ‘pred. III’ equals the level at “pred. I.” During diapause, the gene expression level is significantly downregulated when comparing “d. I” with “pred. I.” *Dmhsp70-B* mRNA expression is downregulated at “d. III,” “d. IV,” and “d. V.” After diapause, *Dmhsp70-B* gene expression is upregulated in comparison with “pred. I” (Figure 2B).

### Gene Expression of *Dmhsp70-A* and *Dmhsp70-B* at a Higher Diapause-Maintenance Temperature (20 ± 0.1°C)

We then examined whether the decreased *hsp70* expression is dependent on the diapause-maintenance temperature. After an incubation period of 30 days at 20 ± 0.1°C, the diapausing embryos show downregulation of *Dmhsp70-A* at “d. III” when compared with “pred. I” embryos, and this level is further decreased at “d. V” compared with “pred. I” (Figure 2C, dotted line). *Dmhsp70-B* expression is significantly downregulated at “d. III” and “d. V” in comparison with “pred. I” (Figure 2D, dotted line). Similar to the embryos stored at 4 ± 0.1°C, both *Dmhsp70-A* and *Dmhsp70-B* decrease during diapause when stored at 20 ± 0.1°C. The expression of both *hsp70*s decreases with the length of diapause when stored at 4 ± 0.1°C, which is not the case in embryos stored at 20 ± 0.1°C.

### Comparison of Asexually Produced Embryos and Sexually Produced Embryos

Based on the cell number and morphology, the gene expression of *Dmhsp70-A* and *Dmhsp70-B* in diapause destined sexually produced embryos was compared with continuously developing asexually produced embryos. In comparison with “stage I” asexually produced embryos, the *Dmhsp70-A* mRNA level is stable at the early pre-diapause stage (“pred. I”). In “pred. III” embryos, the expression level of *Dmhsp70-A* is upregulated in comparison with “stage II” asexually produced embryos. During diapause, *Dmhsp70-A* gene expression is continuously at an increased level in comparison with “stage II” asexually produced embryos. In reactivated embryos, gene expression is significantly lower or levels with the gene expression in the comparable embryonic stages of asexually produced embryos (Figure 2E). During the pre-diapause stage, *Dmhsp70-B* gene expression is significantly downregulated in “pred. I” embryos in comparison with “stage I” asexually produced embryos and shows a downregulation in “pred. III” embryos when compared with “stage II” asexually produced embryos. In comparison with “stage II” asexually produced embryos, *Dmhsp70-B* mRNA shows a decreased expression level in all four diapause stages. After diapause, gene expression of *Dmhsp70-B* in sexually produced embryos levels with the expression in the comparable embryonic stages of asexually produced embryos (Figure 2E). In summary, more *Dmhsp70-A* mRNA and less *Dmhsp70-B* mRNA are detected before and during diapause.

### Distribution of HSP70B Protein During Embryonic Development in *D. magna* Asexually Produced Embryos

**FIGURE 3:**
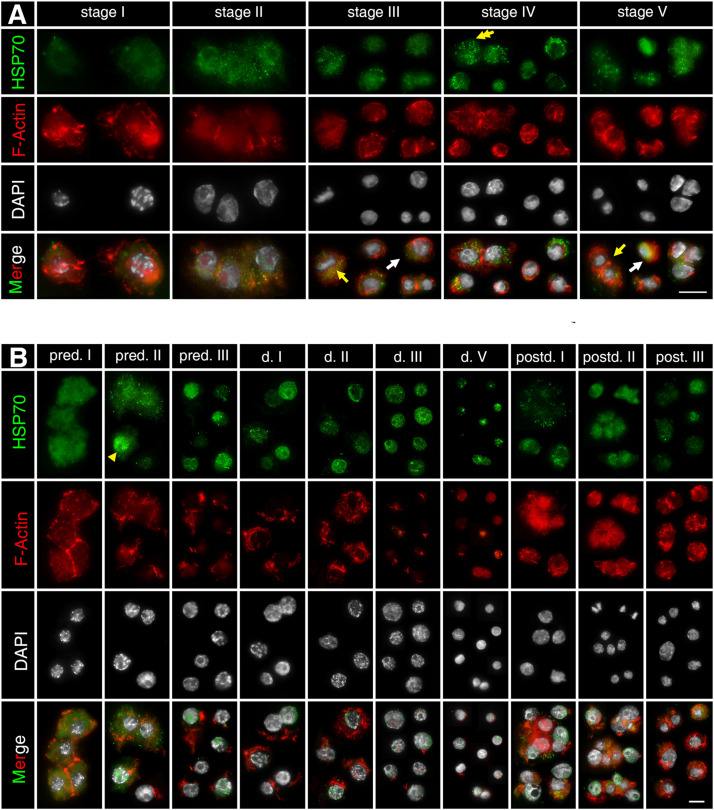
Subcellular localization of HSP70B protein during development of *D. magna* asexually **(A)** and sexually **(B)** produced embryos. Actin filaments were stained with phalloidin in red and chromatin with DAPI in white. **(A)** In developing asexually produced embryos, HSP70B protein (green) distributed homogeneously throughout cytosol and was found in all embryonic stages. HSP70B signals were found in both interphase cells (white arrow) and M-phase cells (yellow arrow). Protein signals at “stage I” were more homogeneously distributed. From “stage II” onward, HSP70B was detected in a granular-like pattern in most cells (yellow double arrow). **(B)** During development of diapause destined embryos, fully developed actin microfilament networks were detected, whereas HSP70B started accumulating in nucleus at “pred. II” (yellow arrowhead). Upon “pred. III,” cytosolic HSP70B vanished. During diapause stage, actin microfilament networks began to disintegrate; HSP70B signals persisted in nuclear area. During post-diapause stage, when embryos were resurrected from diapause, actin microfilament networks rebuilt, accompanied by dissipation of nuclear HSP70B protein and recurrence of cytosolic HSP70B protein. Scale bars = 10 μm.

Asexually produced embryos show expanded actin microfilament networks around the nucleus, which is stained with phalloidin. The actin microfilament networks indicate the form and size of the cytosol (displayed in red, Figure 3). HSP70B protein is detected in all stages in asexually produced embryos (displayed in green, Figure 3). The protein is distributed homogeneously throughout the cytosol. HSP70B-specific immunocytochemical signals are found in both interphase cells (Figure 3A, white arrow) and M-phase cells (Figure 3A, yellow arrow). The protein signals at “stage I” are more homogeneously distributed. From “stage II” onward, HSP70B is detected in a granular-like distribution within most cells (Figure 3A, yellow double arrow).

### Distribution of HSP70B Protein During Embryonic Development in *D. magna* Sexual Produced Embryos

During the pre-diapause stage in *D. magna* sexually produced embryos (“pred. I/II/III”), the extensive actin microfilament networks are displayed within the cytosol. During diapause stages (“d. I/II/III/V”), actin microfilament networks become condensed around the perinuclear area, indicating a reduced cytosol volume. During post-diapause stages (“postd. I/II/III”), when the embryos are resurrected from diapause and development progresses, actin microfilament networks are rebuilt (Figure 3B). The HSP70B subcellular location at “pred. I” is similar to the “stage I” asexually produced embryos (Figure 3B). In some, but not all cells, the HSP70B signal appears co-localized with the nucleus at “pred. II” (Figure 3B, yellow arrowhead). At “pred. III,” HSP70B protein is no longer detected within the cytosol but limited to the nucleus. The HSP70B signals appear in a granule-like form persisting inside the nucleus. In deep diapausing cells (d. V), HSP70B protein reduces, signals do not cover the entire nucleus area, and dot-scattered protein signals are observed. During post-diapause stages (“postd. I/II/III”), developmental resurrection is accompanied by the dissipation of nuclear HSP70B protein and the recurrence of cytosolic HSP70B protein. The resurrected embryos exhibit a similar expression pattern of HSP70B protein to the comparable stages in asexually produced embryos (“stage III/IV/V”). We then assessed whether *Dmhsp70-A* and *Dmhsp70-B* are stress-inducible and can be induced by temperature stresses during diapause.

### Heat Shock Response in Asexually and Sexually Produced Embryos

**FIGURE 4:**
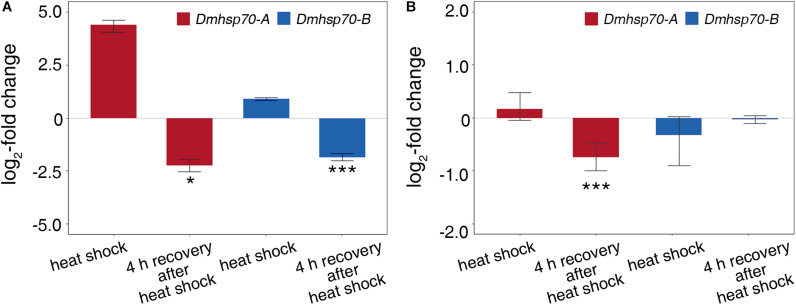
Relative fold change (in log_2_ scale) in transcripts level of *Dmhsp70-A* and *Dmhsp70-B* upon heat shock in developing cells from *D. magna* asexually produced embryos **(A)** and diapausing cells from *D. magna* sexually produced embryos **(B)**. Displayed is expression at each treatment relative to non-stressed control treatment. Error bars represent standard error (*n* = 3). *P*-values are indicated by asterisks: *P ≤ 0.05, ***P ≤ 0.005.

**FIGURE 5:**
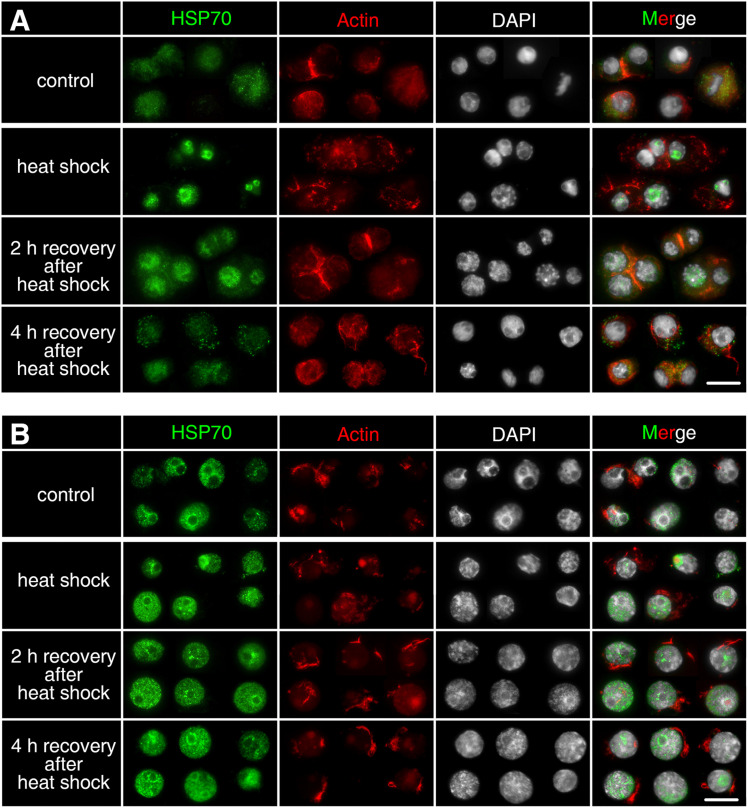
Subcellular localization of HSP70B protein upon heat shock in developing cells from *D. magna* asexually produced embryos **(A)** and diapausing cells from *D. magna* sexually produced embryos **(B)**. Actin microfilament networks were stained with Phalloidin in red and chromatin with DAPI in white. **(A)** In developing cells, HSP70B protein signals localized in nucleus after 1-h heat shock at 33 ± 0.1°C, cytosolic HSP70B protein was strongly reduced. After 2-h recovery at 20 ± 0.1°C, nuclear HSP70B was still detectable, whereas cytosolic HSP70B protein reappeared. After 4-h recovery at 20 ± 0.1°C, HSP70B protein was not detectable in nucleus, a pre-heat homogenous cytosolic distribution was observed. **(B)** One-hour heat shock at 40 ± 0.1°C did not cause distribution changes of HSP70B in diapausing cells. Scale bars = 10 μm.

Comparing with the non-stressed asexual embryos, *Dmhsp70-A* mRNA is not significantly upregulated after 1-h heat shock at 33 ± 0.1°C. After a 4-h recovery at 20 ± 0.1°C, the gene expression is significantly lower than the control group. *Dmhsp70-B* mRNA remains unchanged after the heat shock and shows a significant downregulation after the 4-h recovery when compared with the control group (Figure 4A). In comparison with the non-stressed diapausing embryos, gene expression of *Dmhsp70-A* does not change significantly after 1-h heat shock at 40 ± 0.1°C. After the 4-h recovery at 4 ± 0.1°C, *Dmhsp70-A* is significantly downregulated when compared with the control group. *Dmhsp70-B* mRNA is stabilized after the heat shock and after the 4-h recovery (Figure 4B). After 1-h exposure to 33 ± 0.1°C, changes in the size and form of actin microfilament networks are not observed. HSP70B signals localize in the nucleus area and are especially found around the nucleolar structures. The cytosolic HSP70B protein of the control conditions is strongly reduced in the heat shock condition. After the 2-h recovery phase at 20 ± 0.1°C, nuclear HSP70B protein is still detectable, intense signal around the nucleolus vanishes, and the cytosolic HSP70B protein reappears. After a recovery phase of 4 h at 20 ± 0.1°C, HSP70B protein no longer accumulates in the region of the nucleus; a pre-heat homogenous cytosolic distribution is observed (Figure 5A). Actin microfilament networks are already strongly reduced in diapausing cells; a heat shock at 40 ± 0.1°C has weak effects on the expression of actin microfilament networks. HSP70B protein is predominantly localized in the nucleus of non-stressed diapausing cells. Obvious changes in signal intensity or protein distribution are not found either immediately after heat shock or during the recovery period (Figure 5B).

### Cold Shock Response in Asexually and Sexually Produced Embryos

**FIGURE 6:**
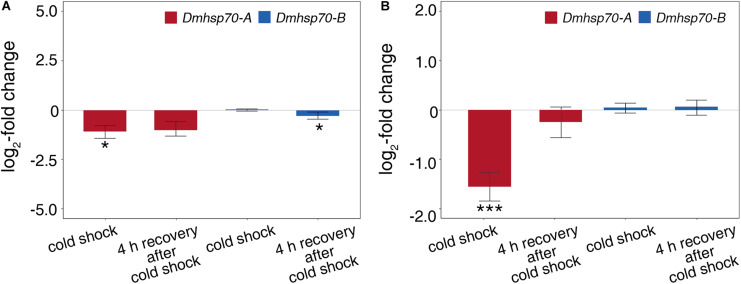
Relative fold change (in log_2_ scale) in transcript level of *Dmhsp70-A* and *Dmhsp70-B* upon cold shock in developing cells from *D. magna* asexually produced embryos **(A)** and diapausing cells from *D. magna* sexually produced embryos **(B)**. Displayed is expression at each treatment relative to non-stressed control treatment. Error bars represent standard error (*n* = 3). *P*-values are indicated by asterisks: *P ≤ 0.05, ***P ≤ 0.005.

**FIGURE 7:**
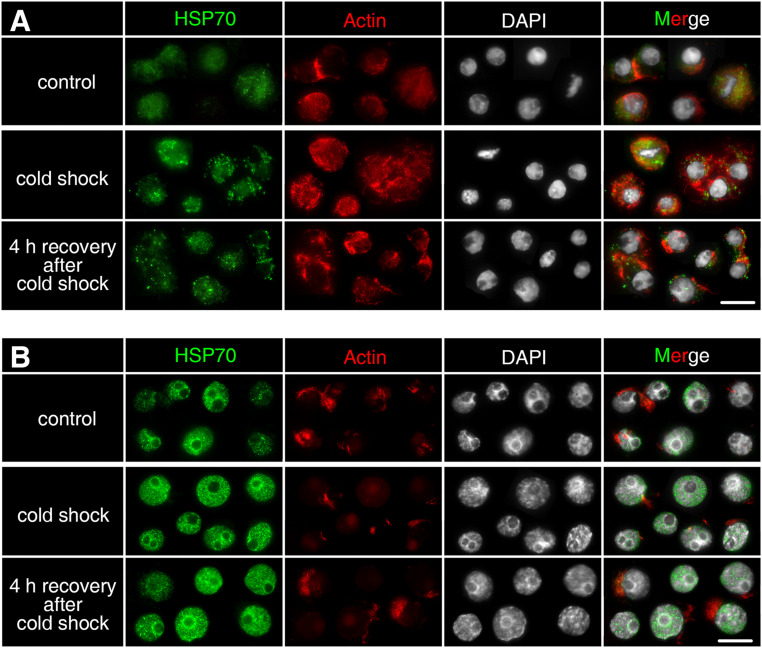
Subcellular localization of HSP70B protein upon cold shock in developing cells from *D. magna* asexually produced embryos **(A)** and diapausing cells from *D. magna* sexually produced embryos **(B)**. Actin microfilament networks were stained with phalloidin in red and chromatin with DAPI in white. One-hour cold shock at 3 ± 0.1°C or –20 ± 0.1°C did not induce distribution changes of HSP70B (green) in **(A)** developing cells or **(B)** diapausing cells. Scale bars = 10 μm.

*Dmhsp70-A* mRNA is downregulated after 1-h cold shock at 3 ± 0.1°C; after 4-h recovery at 20 ± 0.1°C, this level equals the non-stressed control group. Gene expression of *Dmhsp70-B* levels with the expression before the cold shock. After the 4-h recovery, the expression is downregulated (Figure 6A). *Dmhsp70-A* mRNA is downregulated after the 1-h cold shock at −20 ± 0.1°C in comparison with the non-stressed control group. After the 4-h recovery at 4 ± 0.1°C, the gene expression levels with the expression before the cold shock. *Dmhsp70-B* mRNA level does not change after the cold shock and after the 4-h recovery (Figure 6B). The distribution of actin microfilament networks and HSP70B protein is not influenced by cold shock in both embryo types (Figure 7).

## Discussion

**FIGURE 8:**
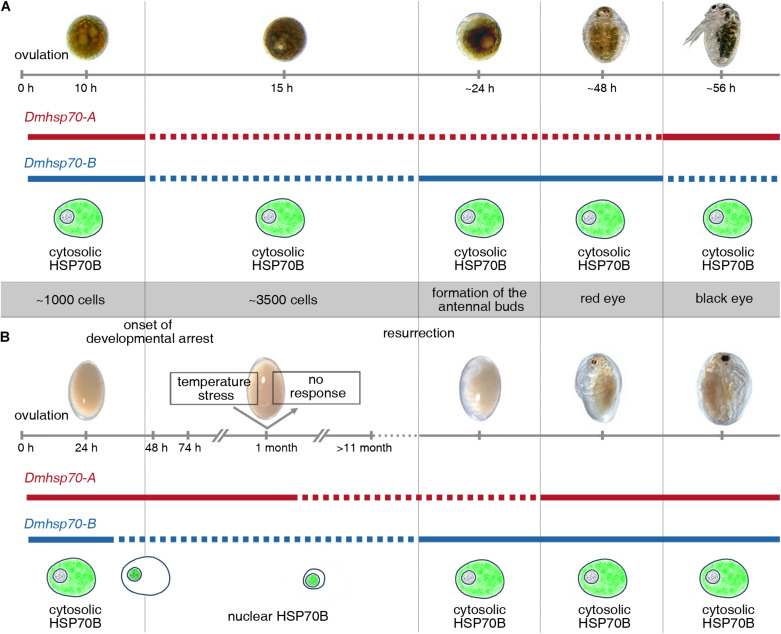
Schematic display of expression patterns of *Dmhsp70-A* and *Dmhsp70-B* and cellular protein distribution of HSP70B (gene product of *Dmhsp70-B*) involved in embryonic diapause of *D. magna*. **(A)** Gene expression analysis in continuously developing asexually produced embryos shows that *Dmhsp70-A* and *Dmhsp70-B* are initially high to become significantly reduced within first 15 h of development and is hypothesized to be a result of use of maternally provided mRNA. Once development progresses, *Dmhsp70-A* expression levels remain downregulated, and *Dmhsp70-B* is significantly upregulated, indicating *Dmhsp70-B* involvement in continuous development. Once embryo reaches late embryonic stage before release from brood pouch, expression patterns of both genes are inverted, indicating that *Dmhsp70-A* is involved in dedicated housekeeping functions. This is further supported by fact that *Dmhsp70-A* expression levels only mildly exceed housekeeping gene expression level. During continuous development, HSP70B is continuously located in cytosol of cells, where it may engage in normal housekeeping functions such as coordination of protein translocations. **(B)** Gene expression analysis in diapause interrupted sexually produced embryos shows that *Dmhsp70-A* is significantly upregulated during diapause preparation and remains high during first phases of diapause (i.e., at least up to 30-day post-ovulation). This indicated *Dmhsp70-A* might be involved in initiating diapause. This is further supported as older embryos 11 months and older expression level significantly declines with time. During this same time, *Dmhsp70-B* is continuously downregulated and stays at a low level. HSP70B is limited to nuclear area cells, where it may engage in cellular clearance mechanisms. Upon resurrection, *Dmhsp70-B* significantly increases in expression but not *Dmhsp70-A*, again pointing to an involvement of *Dmhsp70-B* in continuous development. Just before reaching juvenile stage, both genes are mildly expressed and point to their involvement in general housekeeping functions.

In this study, we examined gene expression changes of two different Hsp70 family members, and subcellular protein location of one of these targets during the development in asexually (continuously developing) and sexually (diapause interrupted development) produced embryos of *D. magna*, as well as their expression under temperature stress (Figure 8). There may be strain-specific differences in the expression patterns of the here investigated Hsp70 family members, but we anticipate that these may be rather minor due to the high conservational degree (with respect to sequence and function) across the animal kingdom (Rosenzweig et al., 2019).

### Function of Hsp70 Family in Embryonic Development

#### *D. magna* Asexually Produced Embryos

To our knowledge, this is the first description of a differential expression pattern of the two investigated *hsp70* genes, *Dmhsp70-A* and *Dmhsp70-B*, during *D. magna* embryogenesis. We observe a continuous expression of both genes right after ovulation. In fact, in this stage, gene expression of both Hsp70 members is larger than in the subsequent “stage II” (see Supplementary Figure 2), indicating that the embryos could be equipped with maternal RNA of both genes. We have previously established that zygotic transmission occurs 16-h post-ovulation (Chen et al., 2018); therefore, the steep decrease of both *hsp70* genes that we observe in “stage II” embryos may be explained by the rapid early embryonic cell cycle, where there is no transcription due to the lack of the two gap phases (G_1_ and G_2_). During this early developmental stage, maternally inherited RNA might be utilized, whereby mRNA levels might decrease as they are being translated into proteins. After “stage II,” *Dmhsp70-A* and *Dmhsp70-B* mRNA expression increases but do not become higher than “stage I” until “stage V” *Dmhsp70-A* and *Dmhsp70-B* have similar expression patterns. Only then *Dmhsp70-A* becomes upregulated, whereas *Dmhsp70-B* becomes downregulated. This implies that *Dmhsp70-A* could be relevant for *D. magna* after embryogenesis but not *Dmhsp70-B*.

The proteins of the different members of the Hsp70 family have been suggested to be involved in certain housekeeping mechanisms of embryogenesis, for instance, by moderating cell cycle, preventing apoptosis, and protecting embryos against environmental stress (Angelier et al., 1996; Dix et al., 1998; Zhu et al., 2013; Radons, 2016). This mirrors our results, as already from “stage I” we see the continuous presence of the protein encoded by the *Dmhsp70-B* gene. HSP70B protein is located within the cytosol throughout embryonic development.

#### *D. magna* Sexually Produced Embryos

In contrast to the low expression level of continuously developing embryos, embryos destined for diapause show completely different expression patterns of *Dmhsp70-A* and *Dmhsp70-B.* Especially *Dmhsp70-A* mRNA is significantly higher expressed at the late pre-diapause stage (i.e., “pred. III”) in comparison with the early pre-diapause stage (i.e., “pred. I”). This expression level is maintained even upon diapause entrance. The involvement of *hsp70* gene products in the preparation and early diapause period has also been reported in the killifish *Austrofundulus limnaeus* and discussed to be associated with enhancing environmental stress resistance (Podrabsky and Somero, 2007). This, in fact, was shown in the flesh fly *Sarcophaga crassipalpis*, where the knockdown of *hsp70* transcripts using RNAi reduced cold-hardiness but did not affect diapause itself (Rinehart et al., 2007). The role of *Dmhsp70-A* in *D. magna* and whether it affects occurrence, duration, quality, or temperature tolerance of diapause is a task for future investigations.

With diapause length, the expression level of *Dmhsp70-A* especially and *Dmhsp70-B* becomes lower in comparison with the initial stage post-ovulation. The natural conditions of diapause in *D. magna* often dictate an environmental temperature of 4 ± 0.1°C, which is also why this temperature is used in laboratory experiments. Nevertheless, the embryos can also be stored at 20 ± 0.1°C. We were thus interested in determining whether the decline in transcript level is associated with the different temperature regimes. We find that the decline is independent of temperature but is actually a function of time. So, with prolonged diapause, transcript levels decrease. This has also been observed in *Delia antiqua* diapause, where *hsp70* mRNA decreased with the diapause duration (Chen et al., 2006).

When the embryonic development resumes upon the exposure to resurrection conditions (“postd. I”), *Dmhsp70-A* transcript level is initially still low in comparison with ‘pred. I’. It is noteworthy to mention that in this case, the embryos used for hatching were 30 days old; the *Dmhsp70-A* level at stage “postd. I” is even lower than the expression at ‘d. III’ (30 days diapause) and equals with the “d. V” (> 11-month diapause). This finding suggests a dedicated downregulation of *Dmhsp70-A* at the beginning of the onset of embryogenesis. In fact, this is in line with the downregulation of this transcript during the embryogenesis of continuously developing embryos. Only at the end of embryogenesis (i.e., “stage V” or “postd. III”) the expression level of this transcript increases and becomes higher than “stage I” or stage “pred. I,” respectively. We, therefore, suggest that this observation points to a functional characteristic of this transcript during embryogenesis *per se*.

Compared with *Dmhsp70-A*, *Dmhsp70-B* demonstrates an opposite expression pattern in sexually produced embryos. During the early pre-diapause stage, embryonic development continues, which is confirmed by the increased cell number and active mitotic activity. Upon entry into a late pre-diapause stage, development slows down, and mitotic activity comes to a halt (Chen et al., 2018). In line with this, *Dmhsp70-B* mRNA gradually declines during pre-diapause and remains low during diapause stages, indicating that it is not involved in diapause preparation or maintenance. Once resurrected from diapause (i.e., “postd. I”), the *Dmhsp70-B* transcript level increases significantly and becomes higher in comparison with the “pred. I.” In the subsequent stages, transcription decreases again in “postd. II” and becomes almost level with “pred. I” in “postd. III.” This may be explained by the function discussed earlier of *Dmhsp70-B* in cellular housekeeping processes.

We underline this hypothesis because the differential expression pattern of *Dmhsp70-B* mRNA coincides with changes in protein distribution. In cells of early pre-diapause staged embryos (“pred. I”), HSP70B distributes in the cytosol. This staining pattern is similar to what we observe in continuous embryogenesis, with the entrance into “pred. II” (44-h post-ovulation), where we see changes in the gene expression pattern, also the protein staining pattern changes. HSP70B accumulates in close vicinity to the nucleus (or even in the nucleus). One of Hsp70’s housekeeping functions is protein translocation. Hsp70 chaperones can bind and transfer newly translated proteins to the outer membranes of the target organelles (such as mitochondria and endoplasmic reticulum) (Rosenzweig et al., 2019). Given the cytosolic distribution of HSP70B in active cells, this Hsp70 member could be relevant for protein translocation inside *Daphnia* cells. However, the global transcriptional level and protein synthesis may already be halted or reduced as diapause entrance approaches. So that in this case, massive protein transfers are not required and explain the absence of cytosolic HSP70B signals. In line with this, we previously discussed that protein translocation processes are reduced due to the lack of microtubule-nets in “d. III” diapause embryos (Chen et al., 2018). In fact, during this phase, HSP70B protein is limited to the nucleus area. Another important mechanism of Hsp70 is the control of protein quality and homeostasis (Rosenzweig et al., 2019). For example, together with proteasomes, HSP70 participates in the degradation of the damaged proteins and especially those in the nucleus (Hjerpe et al., 2016; Morozov et al., 2017). During the long diapause period, degraded proteins may aggregate, which need to be cleared to maintain cell viability. It is thus plausible to suggest that the nuclear HSP70B protein may be part of the clearance mechanism. With age, a severe reduction of the HSP70B protein level is observed in “d. V” diapausing embryos (> 11 months). This decay is not reflected in the hatching ability, as aged *D. magna* diapausing embryos (> 11 months) can be successfully hatched (Cambronero and Orsini, 2017). We suspect that once HSP70B disappears entirely, this will significantly affect the embryos’ viability.

### Comparisons Between Diapause-Destined and Directly Developing Embryos

Studying diapause in *Daphnia* has the advantage that we can directly compare equivalent stages of continuous development with representative stages of diapause interrupted development. When comparing gene expression between the embryo types, we again see the dedicated role of *Dmhsp70-A* in diapause embryos. This *hsp70* gene is expressed at significantly higher levels during the pre-diapause and diapause stages in comparison with the same staged continuously developing embryos. Repression of the cell cycle or even DNA synthesis by an accumulation of the Hsp70 family has indeed been verified in insects and cell lines (Feder et al., 1992; Karlseder et al., 1996; Luft and Dix, 1999; Song et al., 2001).

Clearly, these results call for a more thorough investigation of *Dmhsp70-A* on the protein level. Although *Dmhsp70-A* seems to be relevant for diapause development, *Dmhsp70-B* may be involved in normal cellular activities. As *Dmhsp70-B* is significantly low expressed in diapause-associated development, it is *vice versa* significantly higher expressed in continuously developing embryos. This further underlines its putative involvement in housekeeping functions associated with embryogenesis. Interestingly, “pred. I” diapause destined embryos already contain lower *Dmhsp70-B* mRNA level than “stage I” asexually produced embryos, so that if these transcripts are really inherited from the mother, there must be dedicated machinery that is associated with meiosis even before fertilization and ovulation. The expression level of *Dmhsp70-B* is similar between both embryo types during late embryogenesis, which underlines its involvement in mechanisms beyond embryogenesis.

### Temperature-Induced Hsp70 Expression Change

#### Heat Shock Response

Both *Dmhsp70-A* and *Dmhsp70-B* transcripts showed increased transcription (a tendency toward significance) in response to the heat shock in active cells of asexually produced embryos. This kind of heat-induced rapid accumulation of *hsp70* mRNA has also been reported in other organisms such as insects, including *S. crassipalpis*, *Sesamia nonagrioides*, and *Helicoverpa zea* (Rinehart et al., 2000; Gkouvitsas et al., 2009; Zhang and Denlinger, 2010). Due to its structural particularity, *hsp70* can be rapidly expressed when exposed to stress and timely degraded in the absence of stress (Lindquist, 1980; Wu, 1980; Klemenz et al., 1985; McGarry and Lindquist, 1985; Yost and Lindquist, 1986; Petersen and Lindquist, 1989; Feder et al., 1992). After a 4-h recovery phase, the transcript level is even lower than the pre-heat shock level, which we anticipate being a stress-dependent rebound effect before transcription stabilizes again.

*Dmhsp70-A* and *Dmhsp70-B* are both unresponsive to heat shock during diapause. Only *Dmhsp70-A* is downregulated 4 h after heat shock as seen in active cells. As *Dmhsp70-B* level is constant, the observed downregulation of *Dmhsp70-A* is unlikely due to heat-associated mRNA degradation. We hypothesize that the 1-h heat shock may give the diapausing embryos a resurrection signal. Although the embryos were further incubated in darkness at 4 ± 0.1°C after heat shock, the signal may somehow be translated into cellular signals and trigger downstream physiological processes that are not strong enough to break diapause. If *Dmhsp70-A* functions as a diapause promoter, its downregulation could indicate preparation for resurrection. This is validated by the reduced expression level of *Dmhsp70-A* at the early resurrected stage (“postd. I”). *Dmhsp70-B* is not downregulated in diapausing cells during the recovery period as in active cells. Contrary to *Dmhsp70-A*, embryos may maintain the expression level of *Dmhsp70-B*, which may be associated with active cellular activity.

A heat shock-induced cytosol-nuclear translocation of HSP70B was observed in active cells of asexually produced embryos. This kind of nuclear inflow of HSP70 protein has been seen as an important feature of thermotolerance (Hightower, 1991; Stege et al., 1995; Thayer and Mirkes, 1997; Luft and Dix, 1999; Zeng et al., 2004). Accumulation of HSP70 in the nuclear area reduces the heat-induced intranuclear protein aggregation and may participate in the protection of nucleoplasmic DNA or ribosomal DNA (Stege et al., 1995; Kotoglou et al., 2009; Hjerpe et al., 2016). However, the nuclear translocation of HSP70 may hamper important cellular activities. For normal cellular function and cell growth, HSP70 protein needs to be removed from the nuclear site after heat shock (Velazquez and Lindquist, 1984; Zeng et al., 2004). We also observed this pattern of re-localization in the recovery phase after heat shock in *D. magna*.

Although both *Dmhsp70-A* and *Dmhsp70-B* are not upregulated upon heat shock during diapause, the presence of HSP70B protein may already provide effective protection against temperature stress. In diapausing cells, HSP70B protein is found in a stress-defense-like nuclear position before diapause entrance. The nuclear HSP70B protein may contribute to stress tolerance, as discussed earlier. Besides, it may also result in a heat-shock-like suppression of nuclear and cellular activity and thus promote the diapause entrance.

#### Cold Shock Response

*Dmhsp70-A* is downregulated immediately after cold shock in both diapausing and active cells. This is in line with the observation made in liver tissues of the fish *Larimichthys crocea*, where one *hsp70* member (*hsp70*a5.1) was upregulated under heat stress and downregulated under cold stress (Xu et al., 2018).

During the recovery period, the mRNA level of *Dmhsp70-A* returns to the pre-shock level in both active and diapausing cells. We anticipate that diapausing embryos need to maintain a fundamental level of *Dmhsp70-A*, as it may have some essential functions in diapause maintenance. At this point, it is unclear how *Dmhsp70-A* transcription levels can be resumed in such a short time, despite the low metabolic activity during diapause. Expression of *Dmhsp70-B* and its products do not markedly change upon cold shock in both active and diapausing cells. Likewise, a certain *hsp70* gene was upregulated and maintained throughout pupal diapause of *S. crassipalpis* but could not be further induced by heat or cold shock (Rinehart et al., 2000).

## Conclusion

Our finding suggests dedicated functions of two different Hsp70 members during continuous and diapause intermitted development in *D. magna*. Gene expression of *Dmhsp70-A* is upregulated when the cellular activity is low and downregulated when mitotic activity is high. As *Dmhsp70-B* may be involved in normal cellular activities, its expression decreases before diapause entrance and increases when diapause ends. During diapause, the protein encoded by *Dmhsp70-B* behaves in line with a heat shock-induced response and accumulates in the vicinity of the nucleus. This change in protein distribution has the potential to be used as a marker of diapause start and diapause end.

Our study focuses on just two Hsp70 family members identified in *D. magna*. More investigations focusing on further *hsp70* genes and their products will enable a more comprehensive insight into the physiological functions of this special group.

## Data Availability Statement

The original contributions presented in the study are included in the article/Supplementary Material, further inquiries can be directed to the corresponding author.

## Author Contributions

LC, RG, and LW designed the experiments and discussed the data. LC performed the experiments. LW supported the experiments and gene expression analysis. LC and LW drafted the manuscript. All authors contributed and agreed to the final version of the manuscript.

## Conflict of Interest

The authors declare that the research was conducted in the absence of any commercial or financial relationships that could be construed as a potential conflict of interest.

## Footnotes

1 http://frodo.wi.mit.edu/primer3/
