# Supplementary material for: Distinct Gene Expression Patterns of Two Heat Shock Protein 70 Members During Development, Diapause, and Temperature Stress in the Freshwater Crustacean Daphnia magna
Source: Front Cell Dev Biol. 2021 Jul 1;9:692517. doi: 10.3389/fcell.2021.692517 (PMC8281232; doi:10.3389/fcell.2021.692517)
Supplement: Supplementary file 1 [file Data_Sheet_1.docx]

**Supplemental information**

**Table S1:** Hsp70 members in *D. magna*, information comes from wFleabase.

| **GeneID** | **Scaffold** | **Name** | **isoform** |
| --- | --- | --- | --- |
| Dapma7bEVm002643t1 | scaffold02121:529802-532407:- | Heat shock 70 kDa protein cognate | 13 |
| Dapma7bEVm003115t1 | scaffold00024:1105497-1108214:+ | Heat shock 70 kDa protein cognate | 3 |
| Dapma7bEVm003127t13 | scaffold01774:330-2671:+ | Heat shock 70 kDa protein cognate | 35 |
| Dapma7bEVm011406t1 | scaffold01581:1335969-1338645:- | Heat shock 70 kDa protein cognate | 5 |
| Dapma7bEVm018428t6 | scaffold03339:32689-37399:+ | heat shock protein hsp70-12a/ARP2_G16004 | 6 |
| Dapma7bEVm024396t1 | scaffold03310:1-947:+ | heat shock protein hsp70-12a | 0 |

**Table S2:** List of mean Cqs per measured gene and treatment

| embryo type | stage | *Dmhsp70-A* | *Dmhsp70-B* | *L32* | *tbp* |
| --- | --- | --- | --- | --- | --- |
| asexually produced embryo | pred. I | 25.72 | 19.07 | 17.69 | 20.36 |
|  |  | 26.235 | 19.58 | 17.84 | 20.74 |
|  |  | 26.37 | 19.32 | 17.82 | 20.97 |
|  | pred. III | 24.555 | 22.03 | 17.96 | 22.36 |
|  |  | 24.61 | 20.98 | 17.25 | 21.95 |
|  |  | 24.455 | 21.63 | 17.58 | 21.955 |
|  | d. I | 24.04 | 22.43 | 17.99 | 21.42 |
|  |  | 24.15 | 23.09 | 18.3 | 21.235 |
|  |  | 25.25 | 22.85 | 17.96 | 21.44 |
|  | d. III | 25.61 | 21.68 | 17.86 | 21.515 |
|  |  | 26.25 | 21.58 | 18.15 | 21.48 |
|  |  | 26.37 | 21.89 | 17.6 | 21.71 |
|  | d. III (20 °C) | 27.17 | 22.65 | 18.23 | 21.2 |
|  |  | 27.21 | 22.72 | 18.03 | 21.15 |
|  |  | 27.48 | 22.94 | 18.2 | 21.25 |
|  | d. IV | 27.75 | 21.12 | 17.28 | 20.66 |
|  |  | 27.52 | 21.32 | 17.18 | 20.63 |
|  |  | 27.635 | 21.03 | 17.16 | 20.725 |
|  | d. V | 29.76 | 23.44 | 18.61 | 21.64 |
|  |  | 29.96 | 23.39 | 18.59 | 21.55 |
|  |  | 29.9 | 23.24 | 18.59 | 21.26 |
|  | d. V (20 °C) | 27.92 | 23.2 | 17.59 | 21.9 |
|  |  | 27.47 | 22.88 | 17.5 | 21.58 |
|  |  | 27.31 | 22.91 | 17.31 | 21.58 |
|  | postd. I | 27.045 | 17.18 | 16.77 | 20.66 |
|  |  | 28.63 | 17.83 | 16.75 | 21.11 |
|  |  | 26.95 | 16.84 | 17.17 | 20.63 |
|  | postd. II | 26.615 | 18.59 | 17.7 | 22.935 |
|  |  | 27.27 | 17.92 | 16.77 | 20.88 |
|  |  | 25.26 | 18.49 | 17.97 | 21.955 |
|  | postd. III | 27.015 | 20.33 | 19.42 | 22.205 |
|  |  | 25.815 | 19.36 | 18.55 | 21.755 |
|  |  | 26.195 | 19.88 | 18.87 | 21.295 |
|  |  |  |  |  |  |
| asexually produced embryo | stage I | 26.395 | 17.78 | 17.56 | 21.37 |
|  |  | 26.725 | 17.92 | 17.78 | 21.475 |
|  |  | 26.585 | 17.71 | 17.49 | 21.59 |
|  | stage II | 31.765 | 17.85 | 16.24 | 21.57 |
|  |  | 30.405 | 18.02 | 16.42 | 20.965 |
|  |  | 30.115 | 17.72 | 16.27 | 20.64 |
|  | stage III | 25.88 | 16.01 | 15.86 | 20.565 |
|  |  | 26.24 | 16.1 | 15.97 | 20.665 |
|  |  | 26.23 | 16.46 | 16.2 | 20.615 |
|  | stage IV | 28.21 | 18.61 | 17.5 | 21.575 |
|  |  | 27.875 | 18.3 | 17.38 | 21.62 |
|  |  | 28.075 | 18.52 | 17.46 | 21.45 |
|  | stage V | 26.835 | 20.49 | 19.96 | 22.025 |
|  |  | 27.01 | 20.74 | 20.11 | 21.585 |
|  |  | 27.245 | 20.62 | 19.87 | 21.39 |
|  |  |  |  |  |  |
| sexually produced embryo | control | 25.5 | 21.37 | 17.47 | 20.96 |
|  |  | 26.03 | 21.53 | 17.49 | 20.9 |
|  |  | 26.01 | 21.39 | 17.51 | 20.84 |
|  | heat shock | 25.91 | 21.8 | 17.57 | 21.5 |
|  |  | 25.97 | 22.48 | 17.3 | 21.1 |
|  |  | 25.81 | 21.83 | 17.5 | 21.55 |
|  | 4 h recovery after heat shock | 26.67 | 21.54 | 17.36 | 21.28 |
|  |  | 26.39 | 21.24 | 17.15 | 20.77 |
|  |  | 26.61 | 21.22 | 17.01 | 20.85 |
|  | cold shock | 27.24 | 20.9 | 16.73 | 20.58 |
|  |  | 27.21 | 21 | 16.91 | 20.64 |
|  |  | 27.21 | 21.06 | 17.13 | 20.91 |
|  | 4 h recovery after cold shock | 25.87 | 21.26 | 17.58 | 20.77 |
|  |  | 26.12 | 21.15 | 17.36 | 20.87 |
|  |  | 26.16 | 21.43 | 17.44 | 20.69 |
|  |  |  |  |  |  |
| asexually produced embryo | control | 26.89 | 17.09 | 16.38 | 20.54 |
|  |  | 27.45 | 17.07 | 16.31 | 20.91 |
|  |  | 27.44 | 17 | 16.39 | 20.6 |
|  | heat shock | 23.28 | 17.18 | 17.51 | 21.76 |
|  |  | 22.89 | 16.59 | 16.97 | 20.98 |
|  |  | 22.63 | 16.45 | 16.69 | 20.89 |
|  | 4 h recovery after heat shock | 30.68 | 19.96 | 16.96 | 21.1 |
|  |  | 30.31 | 19.86 | 16.85 | 20.92 |
|  |  | 30.42 | 19.83 | 17.16 | 20.94 |
|  | cold shock | 28.81 | 17.18 | 16.55 | 20.99 |
|  |  | 28.81 | 17.22 | 16.51 | 20.84 |
|  |  | 28.51 | 17.36 | 16.63 | 20.87 |
|  | 4 h recovery after cold shock | 28.65 | 17.44 | 16.63 | 20.79 |
|  |  | 28.52 | 17.23 | 15.98 | 21.08 |
|  |  | 28.49 | 17.92 | 16.85 | 21.31 |

**Table S3:** Fold gene expression change of *Dmhsp70-A* and *Dmhsp70-B* and statistical results of sexually and asexually produced *D. magna* embryos.

| **gene** | **Control stage** | **vs.** | **Treatment stage** | **Fold expression** | **standard error** | **95% confidence interval** | **pValue** | **results** |
| --- | --- | --- | --- | --- | --- | --- | --- | --- |
| ***hsp70*s during the embryonic development in sexually produced embryos** | | | | | | | | |
| *Dmhsp70-A* | pred. I | vs. | pred. III | 3.903 | 3.365 - 4.759 | 2.967 - 5.023 | 0.032 | UP |
| *Dmhsp70-A* | pred. I | vs. | d. I | 3.766 | 2.410 - 5.108 | 2.125 - 5.196 | 0.028 | UP |
| *Dmhsp70-A* | pred. I | vs. | d. III | 1.384 | 1.162 - 1.826 | 1.025 - 1.927 | 0.113 |  |
| *Dmhsp70-A* | pred. I | vs. | d. III (20°C) | 0.634 | 0.576 - 0.714 | 0.528 - 0.741 | 0.053 |  |
| *Dmhsp70-A* | pred. I | vs. | d. IV | 0.319 | 0.290 - 0.348 | 0.274 - 0.356 | 0 | DOWN |
| *Dmhsp70-A* | pred. I | vs. | d. V | 0.159 | 0.142 - 0.182 | 0.132 - 0.191 | 0.000 | DOWN |
| *Dmhsp70-A* | pred. I | vs. | d. V (20°C) | 0.499 | 0.465 - 0.560 | 0.412 - 0.571 | 0.048 | DOWN |
| *Dmhsp70-A* | pred. I | vs. | postd. I | 0.319 | 0.179 - 0.479 | 0.158 - 0.506 | 0 | DOWN |
| *Dmhsp70-A* | pred. I | vs. | postd. II | 1.13 | 0.391 - 2.705 | 0.345 - 2.855 | 0.863 |  |
| *Dmhsp70-A* | pred. I | vs. | postd. III | 1.755 | 1.501 - 2.162 | 1.402 - 2.281 | 0.036 | UP |
| *Dmhsp70-B* | pred. I | vs. | pred. III | 0.376 | 0.315 - 0.436 | 0.308 - 0.501 | 0.064 |  |
| *Dmhsp70-B* | pred. I | vs. | d. I | 0.163 | 0.138 - 0.192 | 0.128 - 0.221 | 0 | DOWN |
| *Dmhsp70-B* | pred. I | vs. | d. III | 0.313 | 0.270 - 0.355 | 0.248 - 0.399 | 0.065 |  |
| *DmHsp70 B* | pred. I | vs. | d. III (20°C) | 0.161 | 0.143 - 0.177 | 0.137 - 0.196 | 0.023 | DOWN |
| *Dmhsp70-B* | pred. I | vs. | d. IV | 0.269 | 0.239 - 0.309 | 0.219 - 0.327 | 0.028 | DOWN |
| *Dmhsp70-B* | pred. I | vs. | d. V | 0.142 | 0.128 - 0.160 | 0.128 - 0.161 | 0.000 | DOWN |
| *Dmhsp70-B* | pred. I | vs. | d. V(20°C) | 0.131 | 0.119 - 0.146 | 0.115 - 0.154 | 0.023 | DOWN |
| *Dmhsp70-B* | pred. I | vs. | postd. I | 2.719 | 2.090 - 3.536 | 1.880 - 4.061 | 0.046 | UP |
| *Dmhsp70-B* | pred. I | vs. | postd. II | 2.457 | 1.851 - 3.023 | 1.665 - 3.415 | 0.071 |  |
| *Dmhsp70-B* | pred. I | vs. | postd. III | 1.464 | 1.230 - 1.700 | 1.119 - 1.926 | 0.036 | UP |
| **temperature stresses in diapausing sexually produced embryos** | | | | | | | | |
| *Dmhsp70-A* | control | vs. | heat shock | 1.118 | 0.966 - 1.389 | 0.784 - 1.410 | 0.472 |  |
| *Dmhsp70-A* | control | vs. | 4 h recovery after heat shock | 0.598 | 0.501 - 0.720 | 0.437 - 0.731 | 0 | DOWN |
| *Dmhsp70-A* | control | vs. | cold shock | 0.34 | 0.278 - 0.418 | 0.247 - 0.435 | 0 | DOWN |
| *Dmhsp70-A* | control | vs. | 4 h recovery after cold shock | 0.841 | 0.679 - 1.038 | 0.614 - 1.089 | 0.215 |  |
| *Dmhsp70-B* | control | vs. | heat shock | 0.799 | 0.536 - 1.014 | 0.510 - 1.047 | 0.321 |  |
| *Dmhsp70-B* | control | vs. | 4 h recovery after heat shock | 0.98 | 0.929 - 1.026 | 0.917 - 1.066 | 0.593 |  |
| *Dmhsp70-B* | control | vs. | cold shock | 1.031 | 0.954 - 1.097 | 0.937 - 1.174 | 0.648 |  |
| *Dmhsp70-B* | control | vs. | 4 h recovery after cold shock | 1.043 | 0.929 - 1.149 | 0.884 - 1.190 | 0.591 |  |
| ***hsp70*s during the embryonic development in asexually produced embryos** | | | | | | | | |
| *Dmhsp70-A* | stage I | vs. | stage II | 0.042 | 0.026 - 0.055 | 0.025 - 0.057 | 0.031 | DOWN |
| *Dmhsp70-A* | stage I | vs. | stage III | 0.605 | 0.566 - 0.656 | 0.537 - 0.683 | 0.038 | DOWN |
| *Dmhsp70-A* | stage I | vs. | stage IV | 0.384 | 0.352 - 0.427 | 0.339 - 0.446 | 0.038 | DOWN |
| *Dmhsp70-A* | stage I | vs. | stage V | 1.698 | 1.343 - 2.123 | 1.258 - 2.218 | 0.033 | UP |
| *Dmhsp70-B* | stage I | vs. | stage II | 0.557 | 0.504 - 0.645 | 0.485 - 0.667 | 0 | DOWN |
| *Dmhsp70-B* | stage I | vs. | stage III | 1.225 | 1.129 - 1.328 | 1.061 - 1.343 | 0.056 |  |
| *Dmhsp70-B* | stage I | vs. | stage IV | 0.642 | 0.588 - 0.716 | 0.576 - 0.740 | 0.052 |  |
| *Dmhsp70-B* | stage I | vs. | stage V | 0.405 | 0.362 - 0.486 | 0.343 - 0.503 | 0 | DOWN |
| **temperature stresses in asexually produced embryos** | | | | | | | | |
| *Dmhsp70-A* | control | vs. | heat shock | 20.880 | 17.441 - 24.167 | 16.222 - 25.953 | 0.067 |  |
| *Dmhsp70-A* | control | vs. | 4 h recovery after heat shock | 0.187 | 0.162 - 0.218 | 0.144 - 0.226 | 0.027 | DOWN |
| *Dmhsp70-A* | control | vs. | cold shock | 0.469 | 0.393 - 0.554 | 0.357 - 0.601 | 0.046 | DOWN |
| *Dmhsp70-A* | control | vs. | 4 h recovery after cold shock | 0.53 | 0.424 - 0.668 | 0.387 - 0.749 | 0.070 |  |
| *Dmhsp70-B* | control | vs. | heat shock | 1.776 | 1.680 - 1.862 | 1.639 - 1.961 | 0.084 |  |
| *Dmhsp70-B* | control | vs. | 4 h recovery after heat shock | 0.242 | 0.227 - 0.255 | 0.221 - 0.272 | 0.000 | DOWN |
| *Dmhsp70-B* | control | vs. | cold shock | 1.011 | 0.946 - 1.064 | 0.922 - 1.134 | 0.849 |  |
| *Dmhsp70-B* | control | vs. | 4 h recovery after cold shock | 0.878 | 0.835 - 0.933 | 0.809 - 0.958 | 0.023 | DOWN |
| **asexually produced embryos vs. sexually produced embryos** | | | | | | | | |
| *Dmhsp70-A* | stage I | vs. | pred. I | 1.098 | 1.005 - 1.217 | 0.976 - 1.271 | 0.233 |  |
| *Dmhsp70-A* | stage II | vs. | pred. III | 101.709 | 69.801 - 158.113 | 64.791 - 193.885 | 0 | UP |
| *Dmhsp70-A* | stage II | vs. | d. I | 98.162 | 60.669 - 174.636 | 46.410 - 206.528 | 0 | UP |
| *Dmhsp70-A* | stage II | vs. | d. III | 36.079 | 23.927 - 53.561 | 22.385 - 72.829 | 0 | UP |
| *Dmhsp70-A* | stage II | vs. | d. V | 8.306 | 6.315 - 13.351 | 6.125 - 14.079 | 0 | UP |
| *Dmhsp70-A* | stage III | vs. | postd. I | 0.578 | 0.317 - 0.853 | 0.289 - 0.920 | 0.03 | DOWN |
| *Dmhsp70-A* | stage IV | vs. | postd. II | 3.232 | 1.117 - 7.700 | 0.981 - 8.217 | 0.109 |  |
| *Dmhsp70-A* | stage V | vs. | postd. III | 1.134 | 0.873 - 1.356 | 0.804 - 1.709 | 0.403 |  |
| *Dmhsp70-B* | stage I | vs. | pred. I | 0.325 | 0.294 - 0.360 | 0.276 - 0.372 | 0.036 | DOWN |
| *Dmhsp70-B* | stage II | vs. | pred. III | 0.219 | 0.182 - 0.267 | 0.171 - 0.289 | 0.058 |  |
| *Dmhsp70-B* | stage II | vs. | d. I | 0.095 | 0.080 - 0.118 | 0.072 - 0.127 | 0 | DOWN |
| *Dmhsp70-B* | stage II | vs. | d. III | 0.183 | 0.161 - 0.218 | 0.140 - 0.230 | 0.071 |  |
| *Dmhsp70-B* | stage II | vs. | d. V | 0.157 | 0.143 - 0.180 | 0.124 - 0.185 | 0.028 | DOWN |
| *Dmhsp70-B* | stage III | vs. | postd. I | 0.721 | 0.541 - 0.931 | 0.513 - 1.056 | 0.131 |  |
| *Dmhsp70-B* | stage IV | vs. | postd. II | 1.242 | 0.968 - 1.588 | 0.851 - 1.658 | 0.322 |  |
| *Dmhsp70-B* | stage V | vs. | postd. III | 1.175 | 0.998 - 1.450 | 0.847 - 1.571 | 0.216 |  |


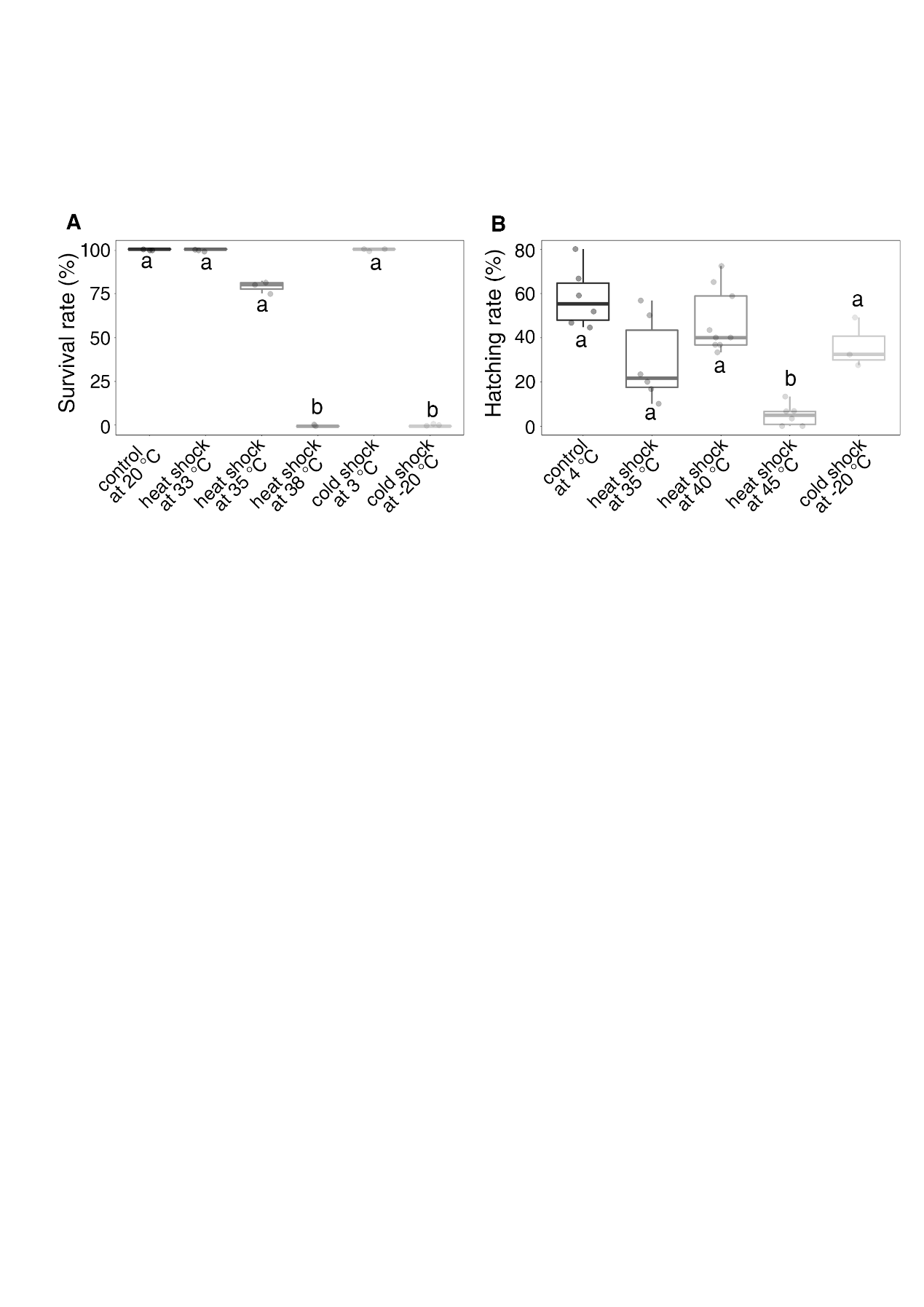


S Figure 1: Survival rate of *D. magna* asexually produced embryos (**A**) and hatching rate of *D. magna* sexually produced embryos (**B**) after exposure to different heat and cold shocks. **A** Pregnant *D. magna* FT442 females with ‘stage III’ asexually produced embryos were exposed to heat/cold shock for one hour, then the females were again incubated at 20 °C ± 0.1 °C. **B** One month old diapausing embryos together with the ephippia were exposed to heat/cold shock. After one hour temperature shock embryos were removed from the ephippia and transferred directly to hatching conditions (24 h light at 23 °C ± 0.1 °C). Points represent the raw data, different letters denote statistically significant differences between groups (*P*≤ 0.05, Kruskal-Wallis pairwise comparison). Survival rate (Kruskal-Wallis test, KW chi-squared = 22.951, df = 5, p = 0.0003449) and hatching rate (Kruskal-Wallis test, KW chi-squared = 19.054, df = 4, p = 0.0007671) was significant different among groups.


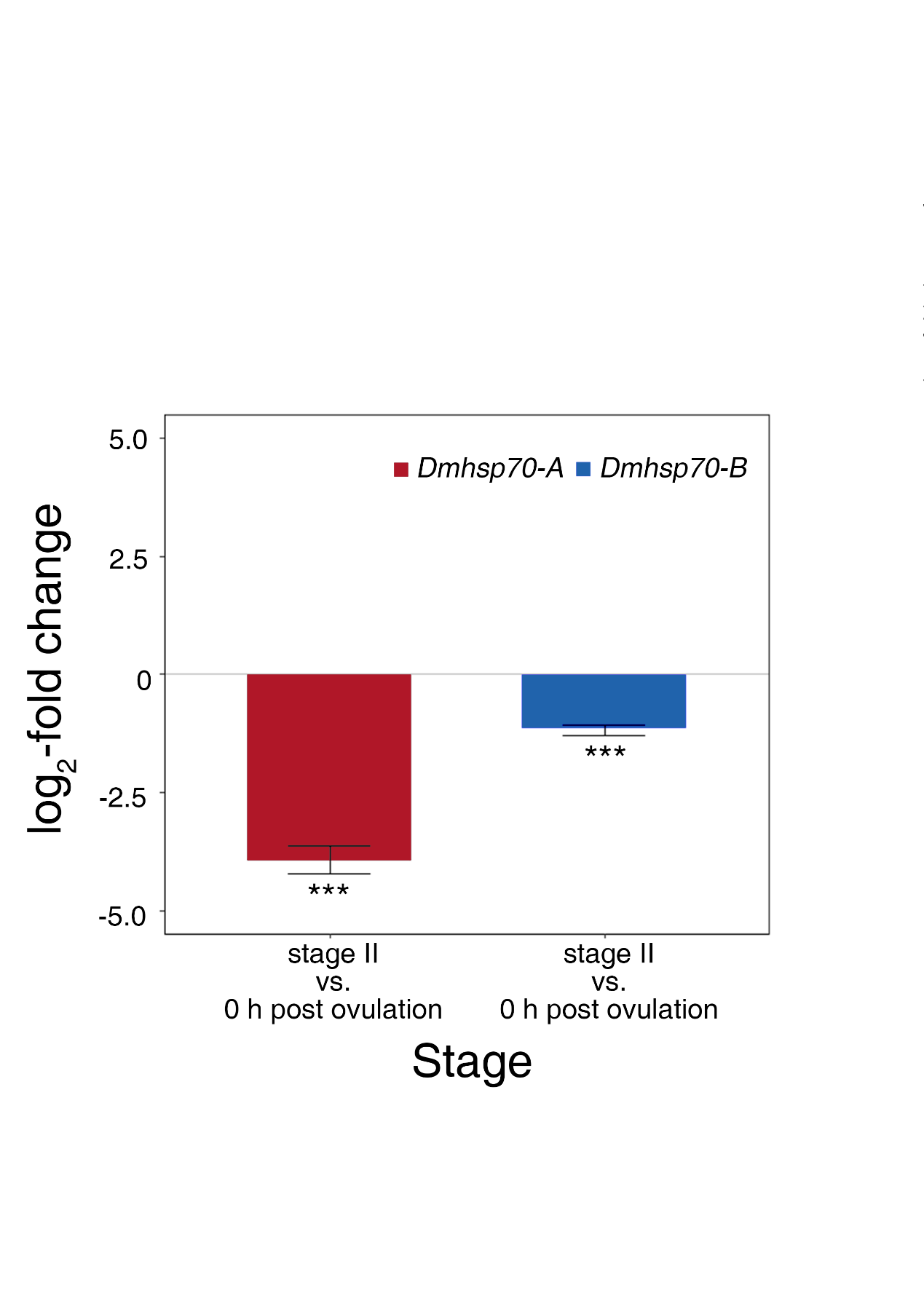


S Figure 2: Expression of *Dmhsp70-A* and *Dmhsp70-B* during early embryonic development in *D. magna* asexually produced embryos. *Dmhsp70-A* and *Dmhsp70-B* decreased significantly at ‘stage II’ in comparison to stage right after ovulations (0 h post ovulation). Error bars represent standard error (n=3). *P* values are indicated by asterisks: *** *P* ≤ 0.005.
